# Supplementary material for: Global survey shows planners use widely varying sea-level rise projections for coastal adaptation
Source: Commun Earth Environ. 2023 Apr 3;4(1):102. doi: 10.1038/s43247-023-00703-x (PMC11041751; doi:10.1038/s43247-023-00703-x)
Supplement: Supplementary file 1 — Supplementary Information [file 43247_2023_703_MOESM1_ESM.pdf]

Supplementary Information for “Global survey shows planners use widely varying sea-level rise projections for coastal adaptation”

Daniella Hirschfeld[1,*], David Behar[2], Robert J. Nicholls[3], Niamh Cahill[4,5], Thomas James[6], Benjamin P. Horton[7,8], Michelle E. Portman[9], Rob Bell[10,11], Matthew Campo[12], Miguel Esteban[13], Bronwyn Goble[14], Munsur Rahman[15], Kwasi Appeaning Addo[16], Faiz Ahmed Chundeli[17], Monique Aunger[18], Orly Babitsky[9], Anders Beal[19], Ray Boyle[20], Jiayi Fang[21], Amir Gohar[22], Susan Hanson[23,3], Saul Karamesines[1], MJ Kim[24], Hilary Lohmann[25], Kathy McInnes[26], Nobuo Mimura[27], Doug Ramsay[28], Landis Wenger[1], Hiromune Yokoki[29]

1. Department of Landscape Architecture and Environmental Planning, Utah State University, 4005 Old Main Hill, Logan, UT 84322-4005, USA
2. San Francisco Public Utilities Commission, San Francisco, CA, USA
3. Tyndall Centre for Climate Change Research, University of East Anglia, Norwich, UK
4. Department of Mathematics and Statistics, National University of Ireland, Maynooth, Ireland
5. Irish Climate Analysis and Research UnitS (ICARUS), Maynooth University, Kildare, Ireland
6. Geological Survey of Canada, Natural Resources Canada, Victoria, Canada
7. Earth Observatory of Singapore, Nanyang Technological University, Singapore, Singapore
8. Asian School of the Environment, Nanyang Technological University, Singapore, Singapore
9. MarCoast Ecosystems Integration Lab, Technion – Israel Institute of Technology, Haifa, Israel 32000
10. Bell Adapt Ltd, Hamilton 3210, New Zealand
11. Environmental Planning Programme, School of Social Sciences, University of Waikato, Te Whare Wananga o Waikato, Hamilton, New Zealand
12. Edward J. Bloustein School of Planning & Public Policy, Rutgers, The State University of New Jersey, New Brunswick, NJ, USA
13. Department of Civil and Environmental Engineering, Waseda University, Tokyo, Japan
14. The Oceanographic Research Institute, Durban, South Africa
15. Institute of Water and Flood Management (IWFM), Bangladesh University of Engineering and Technology (BUET), Dhaka 1000, Bangladesh
16. University of Ghana, Accra, Ghana
17. School of Planning and Architecture, Vijayawada, Andhra Pradesh, India
18. Geological Survey of Canada, Lands and Minerals Sector, Natural Resources Canada 601 Booth Street, Ottawa, ON Canada
19. Woodrow Wilson International Center for Scholars, Washington, DC, USA
20. College of Environmental Design, University of California Berkeley, Berkeley California, USA
21. Institute of Remote Sensing and Earth Sciences, School of Information Science and Technology, Hangzhou Normal University, Hangzhou 311121, China
22. University of the West of England, Bristol, UK
23. Faculty of Engineering and Physical Sciences, University of Southampton, Boldrewood Campus, Burgess Road, Southampton, UK
24. Ministry of Oceans and Fisheries affairs, Busan, Republic of Korea
25. Department of Planning and Natural Resources, St. Croix, USVI
26. Climate Change Research Centre, UNSW Australia, Sydney, NSW, Australia
27. Global and Local Environment Co-creation Institute, Ibaraki University, Ibaraki, Japan
28. National Institute for Water and Atmospheric Research, Auckland, New Zealand
29. Department of Civil, Architectural, and Environmental Engineering, Ibaraki University, Ibaraki, Japan

* Corresponding author: Daniella.hirschfeld@usu.edu; Tel.: +1-435-797-5725

Title: Global survey shows planners use widely varying sea-level rise projections for coastal adaptation
Supplementary Tables and Figures

Supplementary Table 1. Number and percent of respondents by continent, region (explained in supplementary Table 2), and nation.

Scale	Geography	Respondents (#)	Respondents (%)
Continent	Africa	10	4.0%
	Asia	39	15.4%
	Europe	31	12.3%
	North America	126	49.8%
	Australia / Oceania	44	17.4%
	South America	3	1.2%
Region	Africa Atlantic Ocean	5	2.0%
	Africa Indian Ocean	2	0.8%
	Baltic Sea	5	2.0%
	Caribbean Islands	5	2.0%
	East Asia	24	9.5%
	Gulf States	1	0.4%
	North America Atlantic Ocean	78	30.8%
	North America Pacific Ocean	42	16.6%
	Northern Mediterranean	6	2.4%
	North and West Europe	20	7.9%
	Pacific Ocean Large Islands	37	14.6%
	Pacific Ocean Small Islands	8	3.2%
	South America Atlantic Ocean	1	0.4%
	South America Pacific Ocean	2	0.8%
	South Asia	7	2.8%
	South-east Asia	2	0.8%
	Southern Mediterranean	8	3.2%
Nation	Antigua and Barbuda	1	0.4%
	Australia	26	10.3%
	Bangladesh	4	1.6%
	Barbados	1	0.4%
	Brazil	1	0.4%
	Canada	26	10.3%
	Chile	1	0.4%
	China	5	2.0%
	Croatia	2	0.8%
	Denmark	2	0.8%
	Egypt	3	1.2%
	Estonia	1	0.4%
	Fiji	1	0.4%
	Finland	1	0.4%
	France	3	1.2%
	Gambia	1	0.4%
	Germany	2	0.8%

Supplementary Table 1 (continued).

Scale	Geography	Respondents (#)	Respondents (%)
**Nation**	India	2	0.8%
	Ireland	2	0.8%
	Israel	5	2.0%
	Italy	2	0.8%
	Japan	5	2.0%
	Kuwait	1	0.4%
	Liberia	1	0.4%
	Macau	1	0.4%
	Mexico	3	1.2%
	Myanmar	1	0.4%
	Namibia	1	0.4%
	Nauru	1	0.4%
	Netherlands	4	1.6%
	New Zealand	10	4.0%
	Niue	1	0.4%
	Norway	1	0.4%
	Palau	1	0.4%
	Papua New Guinea	1	0.4%
	Peru	1	0.4%
	Poland	1	0.4%
	Samoa	1	0.4%
	Singapore	1	0.4%
	South Africa	3	1.2%
	South Korea	13	5.1%
	Spain	2	0.8%
	St. Lucia	1	0.4%
	Sweden	1	0.4%
	The Philippines	1	0.4%
	Togo	1	0.4%
	Trinidad	1	0.4%
	United Kingdom	8	3.2%
	United States	94	37.2%

Supplementary Table 2. Regional definitions as used in Fig 1.

**Coastal Region**	**Countries/coasts**
Africa Atlantic Ocean	Angola, Benin, Cote d'Ivoire, Cameroon, Democratic Republic Congo, Congo, Western Sahara, Gabon, Ghana, Guinea, Gambia, Guinea-Bissau, Equatorial Guinea, Liberia, Morocco (all coast west of Strait of Gibraltar), Mauritania, Namibia, Nigeria, Senegal, Sierra Leone, Togo, South Africa (West of Cape Agulhas)
Africa Indian Ocean	Djibouti, Eritrea, Kenya, Madagascar, Mozambique, Sudan, Somalia, Tanzania - United Republic, South Africa (East of Cape Agulhas)
Atlantic Ocean Small Islands	Bermuda, Cape Verde, Saint Helena, Saint Pierre and Miquelon, Sao Tome and Principe
Baltic Sea	Aaland, Germany (Baltic Sea coast), Denmark (Baltic Sea coast), Estonia, Finland, Lithuania, Latvia, Poland, Sweden
Central America Atlantic Ocean	Belize, Costa Rica (east coast), Guatemala (east coast), Honduras (east coast), Nicaragua (east coast), Costa Rica , Panama (north coast)
Central America Pacific Ocean	Costa Rica (west coast), Guatemala (west coast), Honduras (west coast), Nicaragua (west coast), Panama (south coast), El Salvador
Caribbean Islands	Aruba, Anguilla, Antigua and Barbuda, Bonaire, Saba and Saint Eustatius, Bahamas, Saint Barthelemy, Barbados, Cuba, Curacao, Cayman Islands, Dominica, Dominican Republic, Guadeloupe, Grenada, Haiti, Jamaica, Saint Kitts and Nevis, Saint Lucia, Saint Martin, Montserrat, Martinique, Puerto Rico, Turks and Caicos Islands, Trinidad and Tobago, Saint Vincent and Grenadine, British Virgin Islands
Russian Federation	Russian Federation
East Asia	China, Hong Kong, Japan, South Korea, Macau, North Korea, Taiwan
Gulf States	United Arab Emirates, Bahrain, Iran, Iraq, Kuwait, Oman, Qatar, Saudi Arabia, Yemen
Indian Ocean Small Islands	French Southern Territories, Cocos Islands, Comoros, Christmas Island, Heard Island and McDonald Islands, British Indian Ocean Territory, Maldives, Mauritius, Mayotte, Reunion, Seychelles
North America Atlantic Ocean	Canada (Atlantic coast including all northern coast), Mexico (all east coast), United States of America (east and south coast including Gulf of Mexico)
North America Pacific Ocean	Canada (Pacific coast, British Columbia and Yukon), Mexico (all west coast), United States of America (all west coast including Alaska)

Northern Mediterranean	Albania, Bulgaria, Bosnia and Herzegovina, Cyprus, Spain (south and east coast/Mediterranean), France (south coast/Mediterranean), Georgia, Gibraltar, Greece, Croatia, Italy, Monaco, Moldova, Malta, Montenegro, Romania, Slovenia, Ukraine
North and West Europe	Belgium, Germany (North Sea cost), Denmark (North Sea cost), Spain (north coast/Atlantic Ocean), France (north and west coast/Atlantic Ocean), Faroe Islands, United Kingdom, Guernsey, Greenland, Isle of Man, Ireland, Iceland, Jersey, Netherlands, Norway, Portugal, Svalbard and Jan Mayen
Pacific Ocean Large Islands	Australia, New Zealand, Papua New Guinea
Pacific Ocean Small Islands	American Samoa, Cook Islands, Fiji, Micronesia, Federal State of, Guam, Kiribati, Marshall Islands, Northern Mariana Islands, New Caledonia, Norfolk Island, Niue, Nauru, Pitcairn Islands, Palau, French Polynesia, Solomon Islands, Tokelau, Tonga, Tuvalu, United States Minor Outlying Islands, United States of America, Vanuatu, Wallis and Futuna, Samoa
South America Atlantic Ocean	Argentina, Brazil, Colombia (north coast), French Guiana, Guyana, Suriname, Uruguay, Venezuela
South America Pacific Ocean	Chile, Colombia (south coast), Ecuador, Peru
South Asia	Bangladesh, India, Sri Lanka, Myanmar, Pakistan
South-east Asia	Brunei Darussalam, Indonesia, Cambodia, Lao Peoples Democratic Republic, Malaysia, Philippines, Singapore, Thailand, East Timor, Virgin Islands, U.S., Viet Nam
Southern Mediterranean	Algeria, Egypt, Israel, Jordan, Lebanon, Libya, Morocco (all coast east of Strait of Gibraltar), West Bank and Gaza, Syrian Arab Republic, Tunisia, Turkey
Southern Atlantic Small Islands	Bouvet Island, Falkland Islands, South Georgia and the South Sandwich Islands

Supplementary Figure 1. Sub-national respondents (A) and local respondents (B) and the populations of those locations.

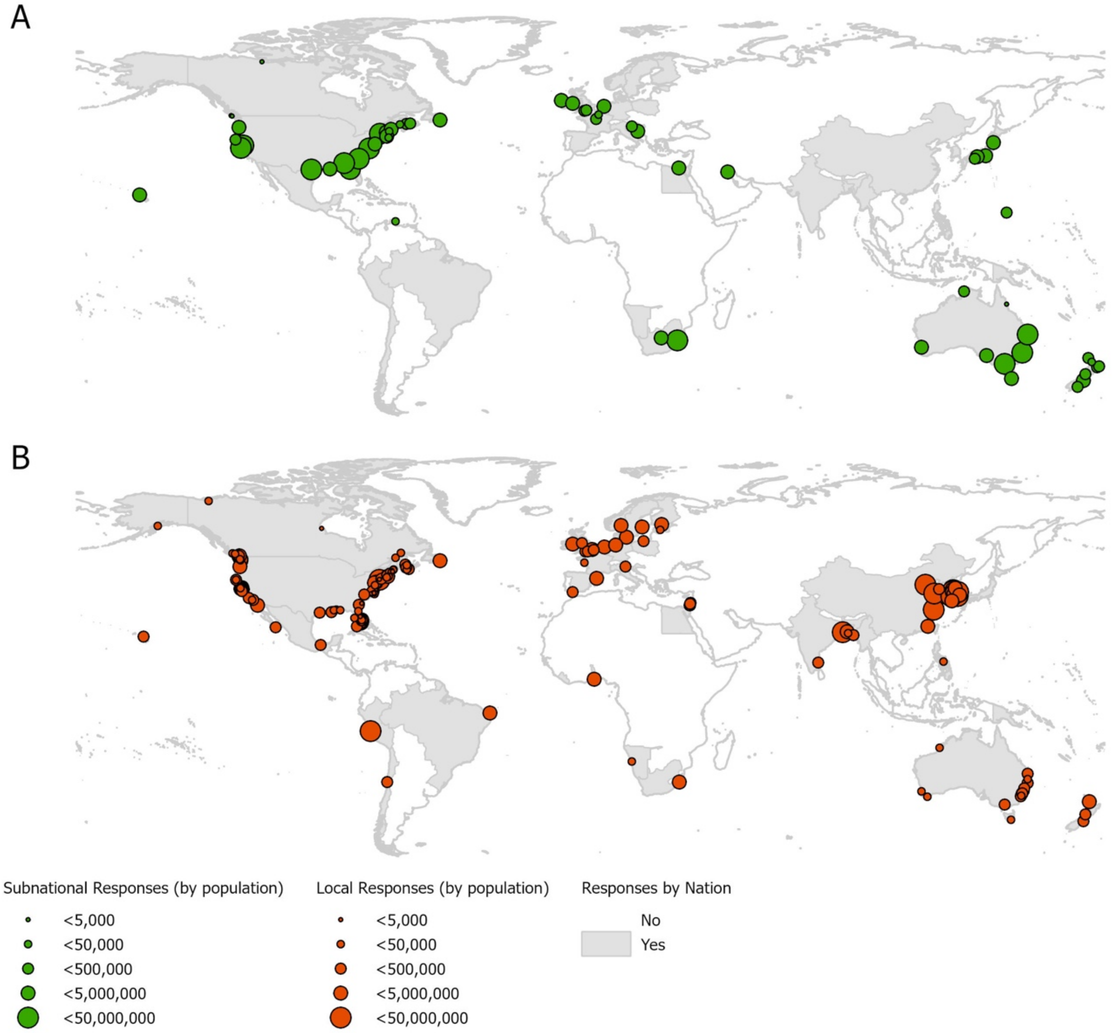

Supplementary Table 3. Number, percent, and population weighting for respondents grouped by degree of sea-level rise projection use in coastal planning. Group 1 are respondents with formally adopted materials that include sea-level rise projections. Group 2 are respondents that are trying to include sea-level rise projections in coastal planning. This group could be at a range of stages from trying to identify the problem to formally launching an assessment. Group 3 are respondents that are not currently working with sea-level rise projections.

Group #	Respondents (#)	Respondents (%)	Population
1	181	72%	745,673,357
2	67	26%	264,186,875
3	5	2%	1,795,195
Totals	253		1,011,655,427

Supplementary Table 4. Number of respondents and percentage by geography at each scale – continent, region, and nation – that are in each of the groups (Table 3).

Scale	Geography	Group 1		Group 2		Group 3	
		#	(%)	#	(%)	#	(%)
Continent	Africa	5	(50%)	5	(50%)		
	Asia	14	(36%)	21	(54%)	4	(10%)
	Europe	27	(87%)	4	(13%)		
	North America	97	(77%)	28	(22%)	1	(1%)
	Australia / Oceania	37	(84%)	7	(16%)		
	South America	1	(33%)	2	(67%)		
Region	Africa Atlantic Ocean	1	(20%)	4	(80%)		
	Africa Indian Ocean	2	(100%)				
	Baltic Sea	5	(100%)				
	Caribbean Islands	1	(20%)	4	(80%)		
	East Asia	9	(38%)	13	(54%)	2	(8%)
	Gulf States	1	(100%)				
	North America Atlantic Ocean	62	(79%)	15	(19%)	1	(1%)
	North America Pacific Ocean	33	(79%)	9	(21%)		
	North and West Europe	19	(95%)	1	(5%)		
	Northern Mediterranean	3	(50%)	3	(50%)		
	Pacific Ocean Large Islands	32	(86%)	5	(14%)		
	Pacific Ocean Small Islands	6	(75%)	2	(25%)		
	South America Atlantic Ocean			1	(100%)		
	South America Pacific Ocean	1	(50%)	1	(50%)		
	South Asia	3	(43%)	3	(43%)	1	(14%)
	South-east Asia	1	(50%)	1	(50%)		
	Southern Mediterranean	2	(25%)	5	(63%)	1	(13%)
Nation	Antigua and Barbuda	1	(100%)				
	Australia	22	(85%)	4	(15%)		
	Bangladesh	2	(50%)	1	(25%)	1	(25%)
	Barbados			1	(100%)		
	Brazil			1	(100%)		
	Canada	21	(81%)	5	(19%)		
	Chile			1	(0.4%)		
	China	2	(40%)	3	(60%)		
	Croatia	1	(50%)	1	(50%)		
	Denmark	2	(100%)				
	Egypt	2	(67%)	1	(33%)		
	Estonia	1	(100%)				
	Fiji	1	(100%)				
	Finland	1	(100%)				
	France	2	(67%)	1	(33%)		

Supplementary Table 4 (continued).

Scale	Geography	Group 1		Group 2		Group 3	
		**#**	**(%)**	**#**	**(%)**	**#**	**(%)**
Nation	Gambia			1	(100%)		
	Germany	2	(100%)				
	India			2	(100%)		
	Ireland	2	(100%)				
	Israel			4	(80%)	1	(20%)
	Italy			2	(100%)		
	Japan	1	(20%)	2	(40%)	2	(40%)
	Kuwait	1	(0.4%)				
	Liberia			1	(100%)		
	Macau			1	(100%)		
	Mexico	1	(33%)	2	(67%)		
	Myanmar	1	(100%)				
	Namibia			1	(100%)		
	Nauru			1	(100%)		
	Netherlands	3	(75%)	1	(25%)		
	New Zealand	9	(90%)	1	(10%)		
	Niue	1	(100%)				
	Norway	1	(100%)				
	Palau			1	(100%)		
	Papua New Guinea	1	(100%)				
	Peru	1	(100%)				
	Poland	1	(100%)				
	Samoa	1	(100%)				
	Singapore	1	(100%)				
	South Africa	3	(100%)				
	South Korea	6	(46%)	7	(54%)		
	Spain	2	(100%)				
	St. Lucia			1	(100%)		
	Sweden	1	(100%)				
	The Philippines			1	(100%)		
	Togo			1	(100%)		
	Trinidad			1	(100%)		
	United Kingdom	8	(100%)				
	United States	76	(81%)	18	(18%)	1	(1%)

Supplementary Figure 2. Respondents (N=143) answered the different ways they apply future SLR projections in their location. Applications included: A) To create a hazards assessment (e.g. in an exposure assessment), B) To frame vulnerability (e.g. in a vulnerability assessment), C) To develop policy, guidance or standards (e.g. in design rules), D)To plan adaptation measures (e.g. in a formal plan), E) To implement adaptation measures (e.g. in a revised zoning code, estimate additional freeboard for defenses, etc.), and F) To design and determine location/height of buildings and infrastructure. For each combination of applications, we show the projection structures of those respondents.

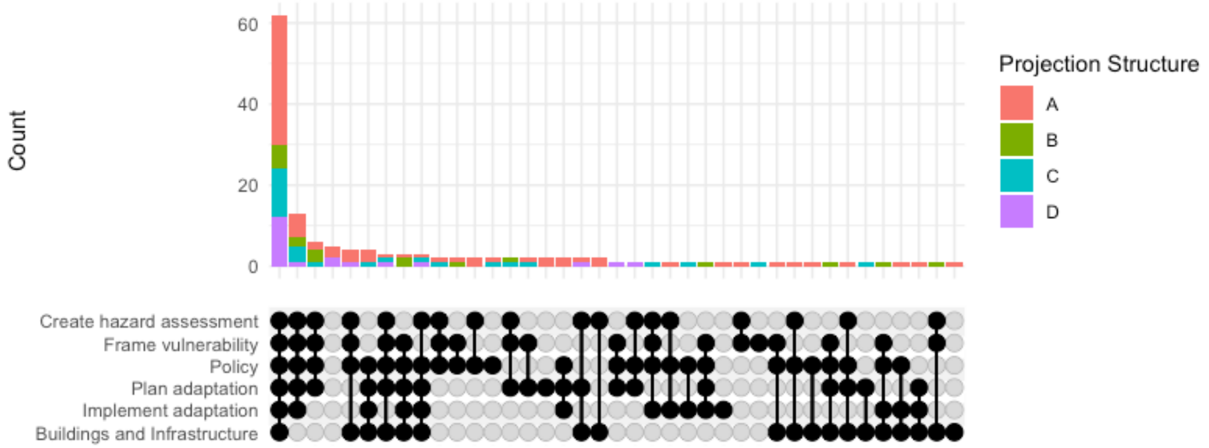

Supplementary Figure 3. Respondents (N=125) answered the criteria they use when doing land-use planning to accommodate different projections. Criteria included: A) The risk profiles of the land uses under consideration, B) The vulnerability of the land uses under consideration, C) The projected costs of the land uses under consideration, D) The planning horizon / planning period of the land use plan, E) Other, and F) No criteria – use the same set of sea-level projections for all our projects. For each combination of criteria, we show the projection structures of those respondents.

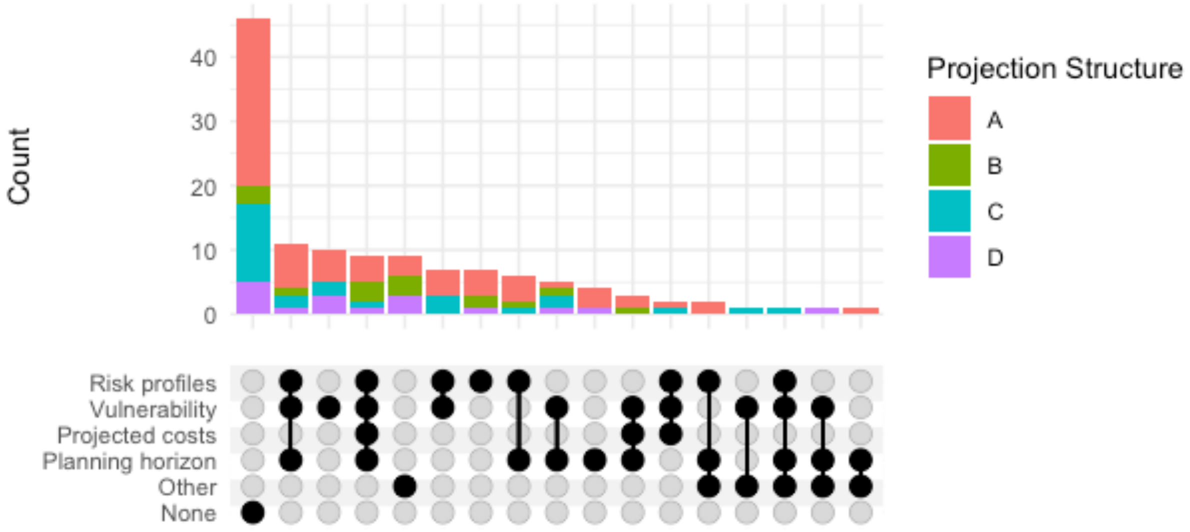

Supplementary Figure 4. Respondents (N=129) answered the criteria they use when doing infrastructure planning to accommodate different projections. Criteria included: A) The risk profiles of the project, B) The projected costs of the project, C) The design life of the project, D) The lifespan of the project, E) Other, and F) No criteria – use the same set of sea-level projections for all infrastructure projects. For each combination of criteria, we show the projection structures of those respondents.

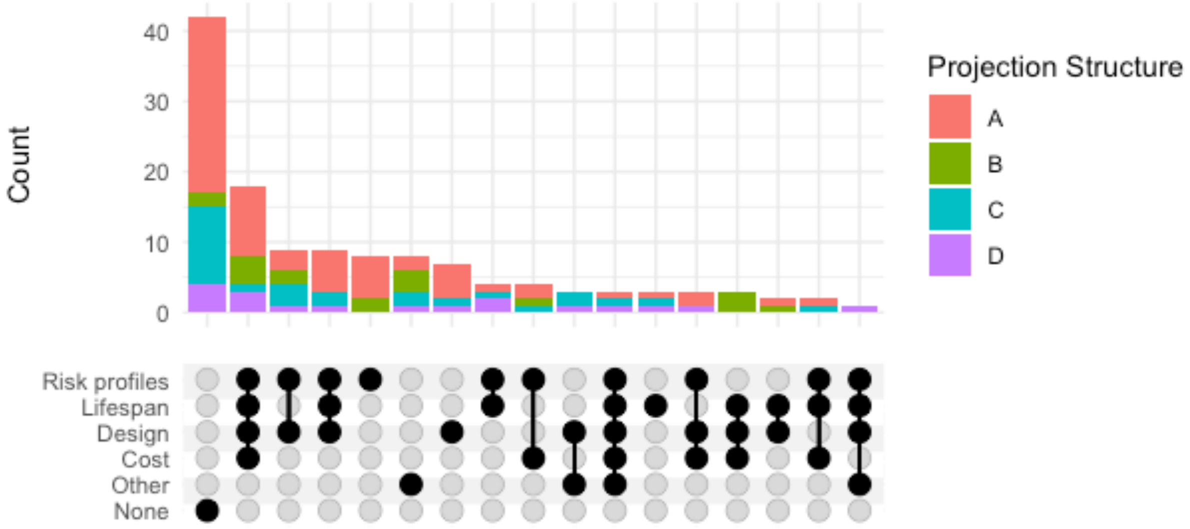

Supplementary Figure 5. Respondents (N=143) structure the use of sea-level rise projections for planning purposes in four ways: A is a singular estimate, B is a low and a high estimate, C is a low, intermediate, and high estimate, and D is a low, intermediate, high, and high-end estimate. Shown are aggregated responses for coastal regions, large nations, and the globe.

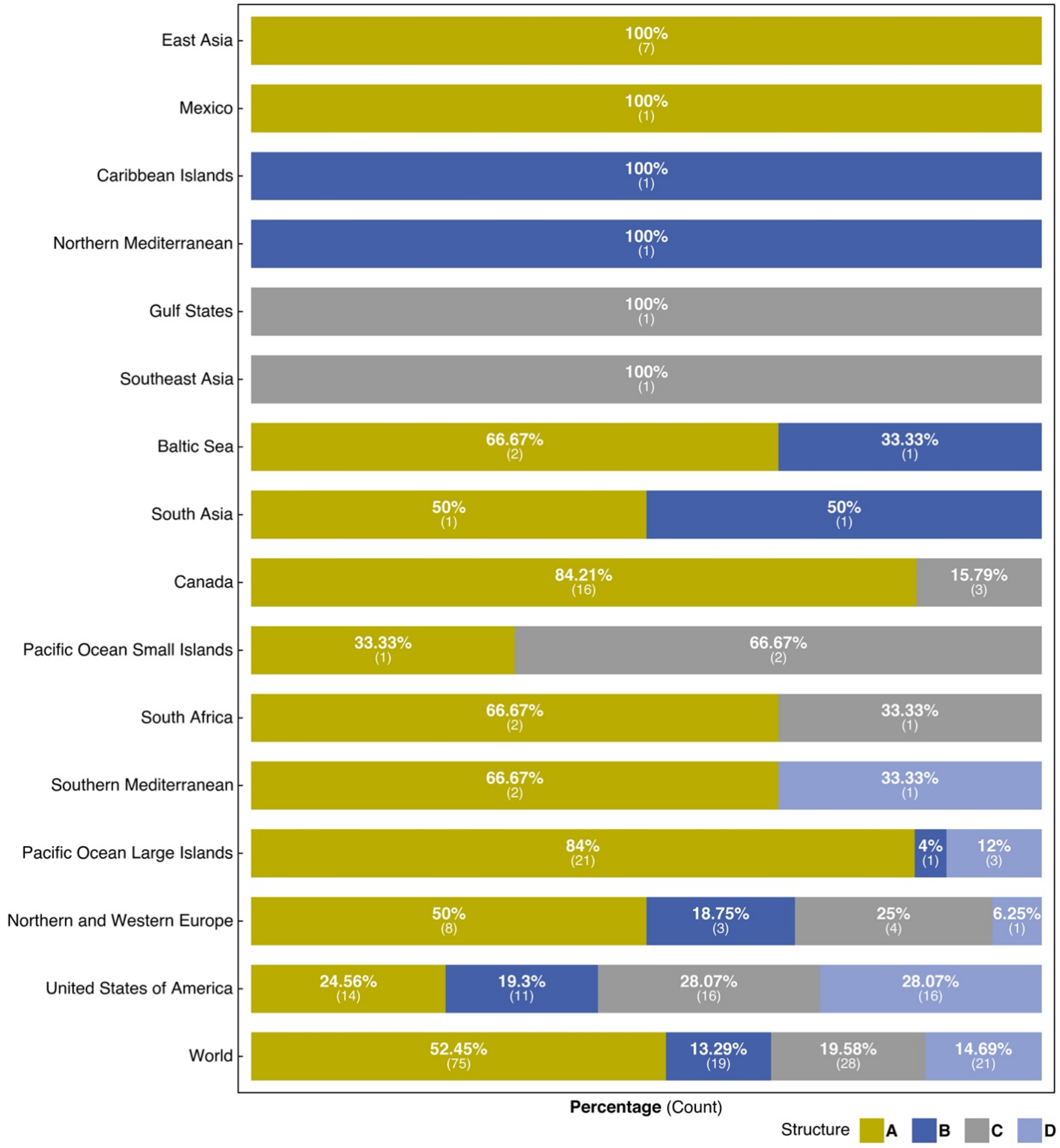

Supplementary Table 5. Summary of the data in Fig 3. Sea-level rise projections in 2100

Structure	Scenario	Min	Max	Median	Mean	n
A	Estimate	0	203.20	90.00	83.74	71
B	Low	7	121.92	60.96	59.95	19
B	High	60	203.20	137.16	140.25	19
C	Low	-10	193.04	41.50	50.51	26
C	Inter	0	187.96	70.79	74.96	26
C	High	0	203.20	115.92	120.71	26
D	Low	0	165.10	53.00	65.20	19
D	Inter	0	198.12	119.38	112.32	19
D	High	0	304.80	190.50	174.55	19
D	Upper	0	304.80	304.80	225.69	19

Supplementary Table 6. Summary of the data in Supplementary Fig 3. Sea-level rise projections in 2050

Structure	Scenario	Min	Max	Median	Mean	n
A	Estimate	-31	100.00	40.00	35.47500	76
B	Low	0	91.44	19.50	21.21400	20
B	High	0	182.88	41.59	43.10200	20
C	Low	0	50.00	20.66	18.23357	28
C	Inter	0	80.00	29.21	31.24214	28
C	High	0	152.40	44.45	49.89571	28
D	Low	-1	76.20	23.00	26.63368	19
D	Inter	0	76.20	43.18	39.80316	19
D	High	0	88.90	60.96	55.61789	19
D	Upper	0	104.14	76.20	56.79067	19

Supplementary Figure 6. SLR projections (in meters) for 2050 provided by respondents that they are using in their coastal plans and guidance documents grouped by the four projection structures (A to D) shown in Fig 2.

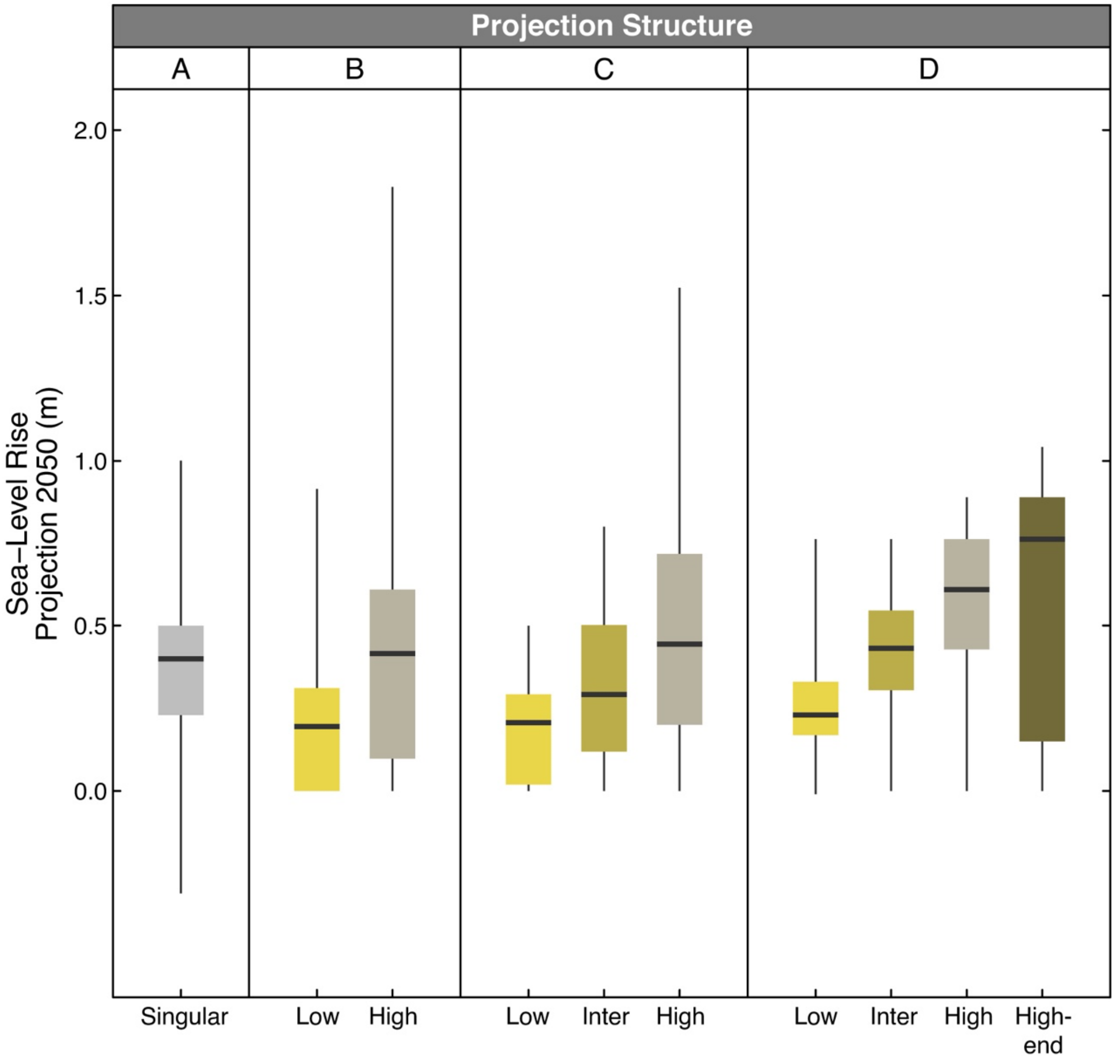

Supplementary Table 7. Number and percent of responses that are above the upper bound of the IPCC projections (RCP8.5 scenario (0.98 m)).

Scenario	# Greater IPCC	N	%
Estimate	28	72	38.8
Low	8	64	12.5
Inter	21	43	48.8
High	47	64	73.4
Upper	15	19	78.9

Supplementary Figure 7. Sources of SLR projections as provided by survey respondents. This represents 177 responses to this question where respondents were able to list multiple sources.

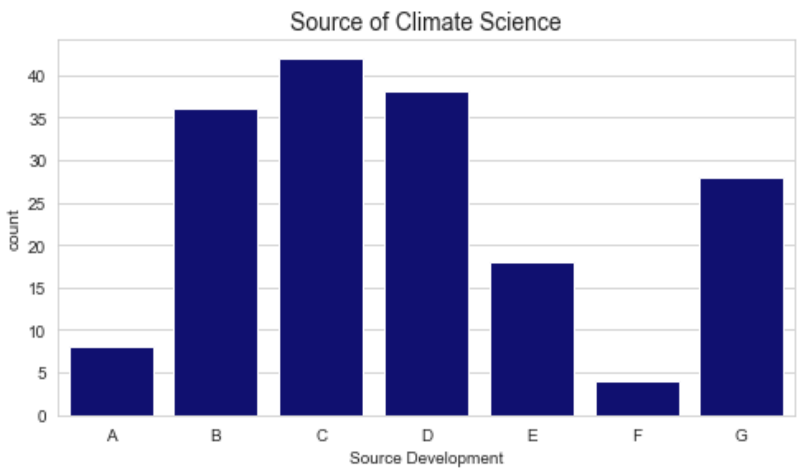

A: Academic Papers
B: Selected from projections
C: Co-produced
D: Generated as guidance by authority
E: Consultants
F: Mandated by authority
G: Other

Appendix 1

Sea Level Projections in Coastal Planning Questionnaire

Welcome

Thank you for participating in our questionnaire on the use of projected sea level change in coastal planning. Sea levels are rising globally due to climate change and are projected to rise at an increasing rate. Across most of the globe, relative sea-level is also rising, while some regions are projected to experience continuing sea-level fall. Many localities are taking action to prepare for these projected changes to sea level. However, not all places and not all planners have access to the same information on future sea levels. Also, localities are using this information in different and unique ways.

We are interested in how different places around the globe are using information on future sea levels in their coastal planning processes. To understand this, we aim to gather information on the current policies or guidance materials in use for your location.

We expect that if you are familiar with your location's policy or guidance, or have access to the appropriate information, the questionnaire will take between 10 and 25 minutes. We have provided a definition document to clarify any unfamiliar terminology.

Questionnaire results will be reported by each specific location (i.e. nation / state / province / territory / district / city / etc.), not by individual respondents. Therefore, the Utah State University's Institutional Review Board approved the study as a non-human subjects study. Your answers will not be associated with your personal information in any way.

Thank you for taking the time to participate in our research!

Useful information for taking this questionnaire

Our goal is to make this questionnaire relatively easy for you to complete. This PDF version is designed to support you in taking the online questionnaire. We recommend you use it in two ways:

1. To familiarize yourself with the questions before you begin answering the questions online. Being familiar will make it faster and easier to enter the information online.
2. To collaborate with other colleagues and make sure that you have the correct answers. If you need, you can circulate the full document, or you can ask a colleague to answer one or two specific questions.

Note: Text in **green** refers to instructions or information that is automated in the online environment. For example, questions that say "Select only one" are questions that the online tool will not allow you to pick multiple answers.

Note: Terms shown in blue with an underline contain a definition and are listed in definitions section starting on page 15.

When you are ready, and have gathered the necessary materials, please go to the online questionnaire with the URL provided in the email.

Thank you for taking the time to participate in our research!

Title: Global survey shows planners use widely varying sea-level rise projections for coastal adaptation
Appendix A: Questionnaire Used

Part 1: General Information

This section of the questionnaire is about your location: where it is and whether future sea levels are considered in coastal planning.

Note: Respondents will only be responsible for one of the next two questions. They will automatically be assigned based on our knowledge.

1A. What location (i.e. city, state, province, territory, region etc.) do you represent?

1B. In which nation is your location? (Select from list)

2. Does your location have guidance materials, reports, or policy documents currently in use that address future sea levels in coastal planning processes? (select only one)

a. Yes (→ continue on next page)
b. No (→ move to "Part 2: Sea-level Planning Constraints" section on page 12)

Part 2: Current guidance or policy for future sea levels in coastal planning

This section of the questionnaire is about the currently used guidance, policy materials, or reports related to future sea levels for planning in your location. We want to know how the information was developed, what the materials say, and how they are applied. Additionally, we would like, if possible, to get a copy of the current most relevant policy or guidance source used for your location.

3. What is the title of the primary source where your location's future sea levels and associated planning applications are specified?

4. In what year did your location start using the above-mentioned source?

(Select from list of years, 1980 - 2020)

5. How were the **currently used** future sea levels in the above-mentioned source developed?

(select only one)

a. Selected from an academic paper or papers
b. Selected from state/province, regional, national or international projections
c. Co-produced between your location and a consulting scientist, academic institution, or government advisor
d. Generated as guidance by a scientific advisory body or government authority and provided to your location
e. Provided by consultants/contractors
f. Mandated or required for your location by a scientific advisory body or government authority
g. Other (Please explain)

6. Are your location's future sea levels designed as?

(select only one)

a. Requirements that must be followed
b. Strongly encouraged for best practices
c. General guidance based on available information

We would like, if possible, to review the source where your location's future sea levels and associated planning applications are specified. This source should be currently in use within your location. The source should include details about the future sea levels and their uses in your planning processes.

7. How can you provide access to the primary source where your location's future sea levels are specified? (select only one)

a. I will provide a web link (provide link below)
b. I will directly upload the file (goes to 7A)
c. I can only provide the title, but cannot provide direct access
d. I cannot provide access to the source

Note: To upload a file, click "I will directly upload the file" in the question above. An upload field will appear near the bottom of the page.

7A. (If question 7 answer is B) Please upload the file below. If you have multiple files, please create and upload a place.zip file. (file upload)

*We would like to know directly from you what **numbers** are part of your future sea levels as specified in the source you provided. The next three questions are specifically about the future sea levels used for planning purposes in 2050 and 2100. Then we ask about planning beyond the year 2100.*

- *The first question is used to understand the number of future sea levels in use.*
- *The subsequent questions are used to gather specific numbers used for planning purposes.*

If you are not able to provide specific numbers, please select "other" in the next question. This will allow you to provide a written explanation of your future sea levels.

8. How does your location structure its information on future sea levels for planning purposes? (select only one)

a. Singular estimate, scenario or curve (→ Q 9 & 10 design in style A)
b. Low and high estimate (→ Q 9 & 10 design in style B)
c. Low, intermediate, and high estimate (→ Q 9 & 10 design in style C)
d. Low, intermediate, high, and upper estimate (→ Q 9 & 10 design in style D)
e. Other (→ Q 9 & 10 design in style E)

Style A

9. What sea-level value is used for planning purposes for the year 2050 (in centimeters)?
   a. Estimate (Numeric answer)

10. What sea-level value is used for planning purposes for the year 2100 (in centimeters)?
   a. Estimate (Numeric answer)

Style B

9. What sea-level values are used for planning purposes for the year 2050 (in centimeters)?
   a. Low estimate (Numeric answer)
   b. High estimate (Numeric answer)

10. What sea-level values are used for planning purposes for the year 2100 (in centimeters)?
   a. Low estimate (Numeric answer)
   b. High estimate (Numeric answer)

Style C

9. What sea-level values are used for planning purposes for the year 2050 (in centimeters)?
   a. Low estimate (Numeric answer)
   b. Intermediate estimate (Numeric answer)
   c. High estimate (Numeric answer)

10. What sea-level values are used for planning purposes for the year 2100 (in centimeters)?
   a. Low estimate (Numeric answer)
   b. Intermediate estimate (Numeric answer)
   c. High estimate (Numeric answer)

Style D

9. What sea-level values are used for planning purposes for the year 2050 (in centimeters)?
   a. Low estimate (Numeric answer)
   b. Intermediate estimate (Numeric answer)
   c. High estimate (Numeric answer)
   d. Upper estimate (Numeric answer)

10. What sea-level values are used for planning purposes for the year 2100 (in centimeters)?
   a. Low estimate (Numeric answer)
   b. Intermediate estimate (Numeric answer)
   c. High estimate (Numeric answer)
   d. Upper estimate (Numeric answer)

Style E – this is a catch all for anyone who has an entirely different structure from above

9. Describe the approach and sea-level values used for planning purposes for the year 2050 (Text)

10. Describe the approach and sea-level values used for planning purposes for the year 2100 (Text)

11. Does your location consider sea-level change beyond the year 2100 for planning purposes? (select only one)

a. Yes (→ Q 11A & 11B)
b. No (→ Part 3, question 12)

11 A. (If question 11 answer is yes) What year is at the end of the planning timeframe used? (Select from list of years, 2101 - 2200)

11 B. (If question 11 answer is yes) Please describe your location's approach to planning for sea levels beyond 2100 (include specific values and examples, if appropriate). (Open text box)

Part 3: Further information on the science behind future sea levels

In this section of the questionnaire we inquire more deeply about the other effects incorporated into the numerical values of future sea levels in your location. If you are unfamiliar with this science or unclear about the answers, please select the option: "unsure / do not know"

12. Is vertical land movement included in the sea-level values provided in the previous section? (select only one)

a. Yes (→ Q 12A)
b. No (→ Q 13)
c. Unsure / do not know (→ Q 13)

12 A. When vertical land movement is incorporated into the sea-level policies or guidance is there specific reference made to any of the following processes: (check all that apply)

a. Tectonic effects (uplift and subsidence)
b. Glacial isostatic adjustment
c. Land subsidence arising from groundwater withdrawal, sediment compaction, and other effects

13. How does your location's source on future sea levels define baseline, or reference, sea level? (select only one)

a. By a specific year
b. By a specific range of years
c. Baseline sea level is defined in a different way
d. Unsure / do not know

13 A. (If question 13 answer is a) Which year does your location's source use to define baseline sea level?

a. (Select from list of years, 1900 - 2020)

13 B. (If question 13 answer is b) What range of years does your location's source use to define baseline sea level?

a. Start Year (Select from list of years, 1900 - 2020)
b. End Year (Select from list of years, 1900 - 2020)

13 C. (If question 13 answer is c) How does your location's source define baseline sea level?

a. (Text entry, multiple lines)

14. When engaged in coastal planning what hazards does your location consider? (Check all that apply)

a. Flooding (→ Q 14A)
b. Erosion
c. Hurricane, tropical cyclone or typhoon
d. Tsunami
e. Sea-ice reduction
f. Permafrost thaw / degradation
g. Unsure / do not know
h. Other (Please explain)

14 A. (If question 14 answer is A) In determining present and future flood levels, what coastal processes and/or riverine processes does your location consider? (Check all that apply)

a. Freshwater runoff
b. Storm surge
c. Wave setup
d. Wave runup
e. Perigean-spring tides (i.e. King tides)
f. Tidal variation
g. Groundwater levels
h. Climate cycles (e.g. El Niño–Southern Oscillation, Pacific Decadal Oscillation)
i. Unsure / do not know
j. Other (Please explain)

15. When engaged in coastal planning does your location consider any of the following climate change impacts? (Check all that apply)

a. Extreme storms (wind and/or waves)
b. Increased river flooding
c. Increased rainfall intensity/duration
d. Rising groundwater
e. Ocean acidification
f. Higher average temperatures
g. Extreme heat
h. Increases in drought
i. Unsure / do not know
j. Other (Please explain)

Part 4: Application of Sea Level Guidance

This section of the questionnaire is about how your location uses its sea level policies or guidance – what are the criteria under which different projections are used, whether your location uses a specific planning approach, and how frequently the numerical values related to projected sea-level change are updated.

16. In what context today does your location apply future sea levels? (Check all that apply)

a. To create a hazards assessment (e.g. in an exposure assessment)
b. To frame vulnerability (e.g. in a vulnerability assessment)
c. To develop policy, guidance or standards (e.g. in design rules)
d. To plan adaptation measures (e.g. in a formal plan)
e. To implement adaptation measures (e.g. in a revised zoning code, estimate additional freeboard for defenses, etc.)
f. To design and determine location/height of buildings and infrastructure
g. Other (Please explain)

17. How does your jurisdiction apply future sea levels? (Check all that apply)

a. Select the most likely or best-estimate projection?
b. Use the worst case/ upper-estimate projection?
c. Use a range of sea-level projections to stress test projects, plans or policies
d. Other (Please explain)

18. Have your location's future sea level estimates directly altered the design of any development plans to-date? (select only one)

a. Yes
b. No

The next two questions are specific to different types of planning – land use planning and infrastructure planning. If you know about both kinds of planning work, you may answer both questions. However, if you only know about one kind of planning, then answer the question that best aligns with your knowledge area.

19. When it comes to **land use planning**, what criteria does your location use for accommodating different projections? (Check all that apply)

   a. Based on the risk profiles of the land uses under consideration in the plan or policy or strategy
   b. Based on the vulnerability of the land uses under consideration in the plan or policy or strategy
   c. Based on the projected costs of the land uses under consideration in the plan or policy or strategy
   d. Based on the planning horizon / planning period of the land use plan or policy or strategy
   e. Other (Please explain)
   f. None, we use the same set of sea level projections for all our projects

20. When it comes to **infrastructure or asset planning**, what criteria does your location use for accommodating different projections? (Check all that apply)

   a. Based on the risk profiles of the project
   b. Based on the projected costs of the project
   c. Based on the design life of the project
   d. Based on the lifespan of the project
   e. Other (Please explain)
   f. None, we use the same set of future sea levels for all our infrastructure projects

21. Does your location use any of the planning approaches listed below? (Check all that apply)

   a. Adaptation pathways (including Dynamic Adaptive Policy Pathways (DAPP))
   b. Decision scaling
   c. Robust decision-making
   d. Scenario planning
   e. Traditional risk-based analysis
   f. Unknown
   g. Other (Please explain)

21. (If question 21 is a, b, c, or d) If possible, please share a link to an example of this planning approach in use. (Text entry, multiple lines)

22. Approximately how often does your location plan/expect to update the projections it uses?

(Select best answer)

a. 1 – 4 years
b. 5 – 10 years
c. More than 10 years
d. Unknown
e. No plan for updates

Part 2: Sea-level Planning Constraints

(For those who responded “No” to question 2. This section will **not** be completed by those that answered the previous pages of questions)

This section of the questionnaire is about your location’s current coastal planning processes. We are looking to understand the coastal hazards your location includes in coastal planning. We are also looking to understand current efforts to include future sea levels in coastal planning.

3. When engaged in coastal planning what hazards does your location consider? (Check all that apply)

a. Flooding (→ Q 3A)
b. Erosion
c. Hurricane, tropical cyclone or typhoon
d. Tsunami
e. Sea-ice reduction
f. Permafrost thaw / degradation
g. Unsure / do not know
h. Other (Please explain)

3A. (If question 3 answer is a) In determining present and future flood levels, what coastal processes and /or riverine processes does your location consider? (Check all that apply)

a. Freshwater runoff
b. Storm surge
c. Wave setup
d. Wave runup
e. Perigean-spring tides (i.e. King tides)
f. Tidal variation
g. Groundwater levels
h. Climate cycles (e.g. El Niño–Southern Oscillation, Pacific Decadal Oscillation)
i. Unsure / do not know
j. Other (Please explain)

4. When engaged in coastal planning does your location consider any of the following climate change impacts? (Check all that apply)

a. Extreme storms (wind and/or waves)
b. Increased river flooding
c. Increased rainfall intensity/duration
d. Rising groundwater
e. Ocean acidification
f. Higher average temperatures
g. Extreme heat
h. Increases in drought
i. Unsure / do not know
j. Other (Please explain)

5. Where in the process of considering future sea levels is your location currently? (Check all that apply)

a. Identifying the problem
b. Gathering information on the problem
c. Defining the specific problem
d. Dealing with specific gaps in knowledge
e. Working to find a consensus on future sea levels
f. Considering or discussing actions on the problem
g. Launching a formal assessment of the problem
h. Engaging in an assessment process
i. Not considering future sea levels
j. Other (Please explain)

Final question for all respondents.

Please provide any additional information or URLs about your location's future sea levels for planning, policies, and procedures that is needed to clarify your answers. (Text entry, multiple lines)

Conclusion: This completes the questionnaire. Thank you very much for your time!

Sea Level Projections in Coastal Planning
Definitions

Introduction
In our questionnaire on the use of future sea levels in planning, we use terms that may be unfamiliar to you. These terms relate to both natural processes and planning processes. The terms appear in the survey in blue underlined text. This document defines those terms.

- **Adaptation pathways:** Planning approaches that focus on the sequence of events over time and the tipping points necessary to shift paths. Dynamic adaptation policy pathways (DAPP) is a specific adaptation pathways approach.

- **Baseline or reference:** An initial sea level used for comparisons.

- **Co-produced:** The process of understanding and/or developing actionable science through active collaboration between scientists and policymakers.

- **Coastal processes:** The movement of water, sand, and land in coastal settings. This includes storm surge, wave setup, wave runup, tides, climate cycles, and in some cases groundwater.

- **Decision scaling:** A decision-making process that informs planning processes and uses a decision analytic framework to reveal the full range of climate information that is needed to best inform the decision at hand.

- **Dynamic adaptation policy pathways (DAPP):** A specific version of adaptation pathways. In this approach people identify alternative ways forward (pathways), that could, singly or in combination, meet agreed objectives, performance or levels of service, despite deepening uncertainty, while remaining responsive to changes when this might be needed (dynamic). Pathways can be combinations of short-term actions and long-term options, Essential is the specification of a monitoring system with actions to be taken when a specific adaptation threshold is reached, identifying early signals and triggers (decision points) for switching pathways or revisiting decisions, ensuring sufficient lead time for implementation.
    - Qualtrics definition: A specific adaptation pathways planning approach where planners identify alternative planning pathways that could meet agreed objectives.

- **Glacial isostatic adjustment:** The ongoing movement of land once burdened by ice-age glaciers. Land is moving up where glaciers once existed and moving down in adjacent areas where glaciers did not reach.

- **Land subsidence:** The gradual or sudden sinking of the Earth's surface from subsurface movement of earth materials.

- **Lifespan:** The functional working life of a project. It is the full length of time a project will be in use at this location (including regular repair and maintenance). Typically, engineers and planners select a planning horizon aligned with a project's "design life." The design life is the period of time during which the asset or facility is expected to perform within its specified parameters; in other words, the life expectancy of the asset or facility as constructed. However, most structures and facilities are in service at their given locations far beyond their design life as defined above. An asset might have a design life of 30 years, but might in reality be in service for 50-, 75-, or 100-years or more with regular repair or maintenance.

- **Perigean-spring tides** (i.e. King tides): The highest tides occurring 3 or 4 times per year when the gravitational pull of the sun and moon align.

- **Planning approaches**: The method by which a plan is generated, including the selection of models, priorities, and data.

- **Risk profiles:** A quantitative analysis of the types of threats an asset, a project or a place face.

- **Riverine processes:** The movement of water, sediment and land in river settings.

- **Robust decision-making:** A decision-making planning processes which is meant to produce decisions that have satisfactory performance across a large range of plausible futures.

- **Scenario planning:** A decision-making planning process characterized by the generation and examination of several potential alternative scenarios. These scenarios may include both sea level rise and socioeconomic conditions.

- **Tectonic effects:** Movement of the Earth's surface in response to seismic causes.

- **Upper estimate:** The highest future sea level estimate based on extreme but plausible information. It is often referred to as H++.

- **Wave runup:** The maximum vertical extent of wave uprush on a beach or structure above the still water level.

- **Wave setup:** The increase in mean water level due to the presence of breaking waves
