# Supplementary material for: Global survey shows planners use widely varying sea-level rise projections for coastal adaptation
Source: Commun Earth Environ. 2023 Apr 3;4(1):102. doi: 10.1038/s43247-023-00703-x (PMC11041751; doi:10.1038/s43247-023-00703-x)
Supplement: Supplementary file 3 — Reporting Summary [file 43247_2023_703_MOESM3_ESM.pdf]

nature portfolio

Corresponding author(s): DBPR - COMMSENV-22-0539

Last updated by author(s): Jan 30, 2023

Reporting Summary

Nature Portfolio wishes to improve the reproducibility of the work that we publish. This form provides structure for consistency and transparency in reporting. For further information on Nature Portfolio policies, see our Editorial Policies and the Editorial Policy Checklist.

Statistics

For all statistical analyses, confirm that the following items are present in the figure legend, table legend, main text, or Methods section.

n/a | Confirmed

- [ ] [x] The exact sample size (*n*) for each experimental group/condition, given as a discrete number and unit of measurement
- [ ] [ ] A statement on whether measurements were taken from distinct samples or whether the same sample was measured repeatedly
- [ ] [ ] The statistical test(s) used AND whether they are one- or two-sided
  Only common tests should be described solely by name; describe more complex techniques in the Methods section.
- [ ] [ ] A description of all covariates tested
- [ ] [ ] A description of any assumptions or corrections, such as tests of normality and adjustment for multiple comparisons
- [ ] [ ] A full description of the statistical parameters including central tendency (e.g. means) or other basic estimates (e.g. regression coefficient) AND variation (e.g. standard deviation) or associated estimates of uncertainty (e.g. confidence intervals)
- [ ] [ ] For null hypothesis testing, the test statistic (e.g. *F*, *t*, *r*) with confidence intervals, effect sizes, degrees of freedom and *P* value noted
  Give P values as exact values whenever suitable.
- [ ] [ ] For Bayesian analysis, information on the choice of priors and Markov chain Monte Carlo settings
- [ ] [ ] For hierarchical and complex designs, identification of the appropriate level for tests and full reporting of outcomes
- [ ] [ ] Estimates of effect sizes (e.g. Cohen's *d*, Pearson's *r*), indicating how they were calculated

Our web collection on statistics for biologists contains articles on many of the points above.

Software and code

Policy information about availability of computer code

Data collection	*Provide a description of all commercial, open source and custom code used to collect the data in this study, specifying the version used OR state that no software was used.*
Data analysis	*Provide a description of all commercial, open source and custom code used to analyse the data in this study, specifying the version used OR state that no software was used.*

For manuscripts utilizing custom algorithms or software that are central to the research but not yet described in published literature, software must be made available to editors and reviewers. We strongly encourage code deposition in a community repository (e.g. GitHub). See the Nature Portfolio guidelines for submitting code & software for further information.

Data

Policy information about availability of data

All manuscripts must include a data availability statement. This statement should provide the following information, where applicable:

- Accession codes, unique identifiers, or web links for publicly available datasets
- A description of any restrictions on data availability
- For clinical datasets or third party data, please ensure that the statement adheres to our policy

All data is available through Utah State Universities online data archive at this URL: https://digitalcommons.usu.edu/all_datasets/198/

Human research participants

Policy information about studies involving human research participants and Sex and Gender in Research.

Reporting on sex and gender	NA
Population characteristics	NA
Recruitment	We used a snowball sampling approach to reach as many geographic locations as possible. This sampling technique is ideally suited to circumstances where it can be difficult to adequately define the sampling frame. We asked collaborating researchers and climate change specialists at national and regional levels to provide names and contacts at more localized jurisdictions.
Ethics oversight	This study was considered a non human subjects research project by Utah State University IRB under protocol #11115. We are reporting on publicly available data.

Note that full information on the approval of the study protocol must also be provided in the manuscript.

Field-specific reporting

Please select the one below that is the best fit for your research. If you are not sure, read the appropriate sections before making your selection.

☐ Life sciences ☒ Behavioural & social sciences ☐ Ecological, evolutionary & environmental sciences

For a reference copy of the document with all sections, see nature.com/documents/nr-reporting-summary-flat.pdf

Behavioural & social sciences study design

All studies must disclose on these points even when the disclosure is negative.

Study description	This study is a mixed methods study with both qualitative and quantitative data gathered through a survey instrument
Research sample	Coastal managers across the globe. Age & demographic data were not collected as they are not relevant to the study. This sample was chosen as it is the best way to access public policy and planning occurring around the world.
Sampling strategy	We used a snowball sampling approach to reach as many geographic locations as possible. This sampling technique is ideally suited to circumstances where it can be difficult to adequately define the sampling frame. We used all known best practices to improve sample diversity.
Data collection	The questionnaire was conducted via an online survey platform, Qualtrics. The survey was written in English by the authors, several of whom are native speakers. The survey was then translated into 8 languages (Arabic, Chinese, French, Hebrew, Japanese, Korean, Portuguese, and Spanish) by professional translators. Native speakers of each language verified the translations.
Timing	Data was collected starting in November 2020 and continued through August 2021
Data exclusions	We handled some anomalies in the data by switching zeros to NA in cases where zero didn't make sense relative to other values provided. We also re-labelled 3 structures to reflect the data provided.
Non-participation	We had a 38% response rate
Randomization	*If participants were not allocated into experimental groups, state so OR describe how participants were allocated to groups, and if allocation was not random, describe how covariates were controlled.*

Reporting for specific materials, systems and methods

We require information from authors about some types of materials, experimental systems and methods used in many studies. Here, indicate whether each material, system or method listed is relevant to your study. If you are not sure if a list item applies to your research, read the appropriate section before selecting a response.

Materials & experimental systems

n/a	Involved in the study
☒	☐ Antibodies
☒	☐ Eukaryotic cell lines
☒	☐ Palaeontology and archaeology
☒	☐ Animals and other organisms
☒	☐ Clinical data
☒	☐ Dual use research of concern

Methods

n/a	Involved in the study
☒	☐ ChIP-seq
☒	☐ Flow cytometry
☒	☐ MRI-based neuroimaging
